# Biological and Clinical Insight from Analysis of the Tumor B-Cell Receptor Structure and Function in Chronic Lymphocytic Leukemia

**DOI:** 10.3390/cancers14030663

**Published:** 2022-01-28

**Authors:** Francesco Forconi, Stuart A. Lanham, Giorgia Chiodin

**Affiliations:** 1 School of Cancer Sciences, Cancer Research UK and NIHR Experimental Cancer Medicine Centres, University of Southampton, Southampton SO16 6YD, UK; s.a.lanham@soton.ac.uk (S.A.L.); g.chiodin@soton.ac.uk (G.C.); 2 Department of Haematology, University Hospital Southampton NHS Trust, Southampton SO16 6YD, UK

**Keywords:** chronic lymphocytic leukemia, B-cell receptor, surface IgM, immunogenetics, IGHV1-69, IGHV3-21, BTK, ibrutinib, venetoclax

## Abstract

**Simple Summary:**

The goal of this review is to describe the biological and clinical implications associated with the analysis of the immunoglobulin (also known as B-cell receptor) expressed on the surface of the tumor cells of chronic lymphocytic leukemia (CLL). Analysis of the surface immunoglobulin structure, levels, and signaling characteristics has regularly improved our understanding of this leukemia for the last +20 years since the identification of two subsets with unmutated tumor immunoglobulin (U-CLL) and bad prognosis or mutated immunoglobulins (M-CLL) and good prognosis. In this review, the authors summarize how analysis of the tumor immunoglobulin informs origin, maintenance, progression, current therapy choice, and prognosis of CLL while providing clues for future investigations.

**Abstract:**

The B-cell receptor (BCR) is essential to the behavior of the majority of normal and neoplastic mature B cells. The identification in 1999 of the two major CLL subsets expressing unmutated immunoglobulin (Ig) variable region genes (U-IGHV, U-CLL) of pre-germinal center origin and poor prognosis, and mutated IGHV (M-CLL) of post-germinal center origin and good prognosis, ignited intensive investigations on structure and function of the tumor BCR. These investigations have provided fundamental insight into CLL biology and eventually the mechanistic rationale for the development of successful therapies targeting BCR signaling. U-CLL and M-CLL are characterized by variable low surface IgM (sIgM) expression and signaling capacity. Variability of sIgM can in part be explained by chronic engagement with (auto)antigen at tissue sites. However, other environmental elements, genetic changes, and epigenetic signatures also contribute to the sIgM variability. The variable levels have consequences on the behavior of CLL, which is in a state of anergy with an indolent clinical course when sIgM expression is low, or pushed towards proliferation and a more aggressive clinical course when sIgM expression is high. Efficacy of therapies that target BTK may also be affected by the variable sIgM levels and signaling and, in part, explain the development of resistance.

## 1. Introduction

Chronic lymphocytic leukemia (CLL) is the most common leukemia in the adult population of the Western world and typically manifests as an increase in CD5+ve, CD23+ve clonal B cells with low surface immunoglobulin (sIg) expression in the peripheral blood. CLL cells accumulate in other tissues, including lymph nodes and bone marrow. The low sIg expression of circulating CLL cells is a diagnostic feature that distinguishes CLL from other mature B-cell tumors and normal B-cells [1].

The easy access of tumor cells by a simple blood draw and the opportunity to follow patients long term before requiring treatment has facilitated research in CLL. In the peripheral blood compartment, the CLL cells have a phenotype of activated cells that typically express 10 times less sIg than the bulk of normal B-cells [2]. However, there is a proliferating compartment of Ki67+ve CLL cells in the so-called “proliferation centers” or “pseudofollicles” of lymph nodes. These proliferation centers vaguely resemble the secondary follicles reacting to infective agents but have significant differences in the fact that they lack polarization into a dark and light zone and have modest evidence of T cell interaction. The proliferating cells represent a minor component of the total CLL clone, and the outcome is the presence of 0.1–1% new dividing cells exiting in the peripheral blood of CLL patients per day [3,4].

Proliferation facilitates the acquisition of genetic mutations. Some of these, including *13q* deletion, trisomy *12* and *NOTCH1* mutations, are early events of pathogenetic importance, and systematic analyses in large cohorts of patients revealed the potentials of genetic profiling to stratify CLLs into prognostic subsets with different outcomes following immunochemotherapy [5,6]. However, not all CLLs necessarily carry genetic lesions, and many of those lesions are subclonal. Moreover, the same lesions can be identified in normal B cells or low-count monoclonal B-cell lymphocytosis from the general population and, similar to other mature B-cell tumors [7], cannot alone explain cancer transformation [8,9,10].

There is another type of mutation that is not the result of an unrepaired error but rather a completely natural process that has operated in the normal B-cell before transformation into leukemia. These mutations are obtained by somatic hypermutation (10^6^ times above background mutation rate) of the Ig genes (*IG*) of the heavy-chain variable region (*IGHV*), and kappa (*IGKV*) or lambda (*IGLV*) paired variable light chains. This is an active process involving the enzyme activation-induced cytidine deaminase (AID), which is initiated following antigen engagement and occurs in the germinal center to mature affinity to antigens and ultimately fight infection [11,12].

B cells can transform into a tumor at any stage during their differentiation, and analysis of the IG rearrangements can define that stage [13]. The observation that CLL arises from B cells with unmutated or mutated *IG* and that *IG* status of the CLL clone informs patient’s prognosis [14,15] has opened a long series of investigations on the meaning of B-cell receptor (BCR) structure and function in CLL, ultimately leading to the development of a class of drugs, commonly called BCR-associated kinase inhibitors, which are now dominating the therapeutic scene for patients with CLL.

This review summarizes the clinical significance of BCR structure and function in CLL, which has provided insight into novel therapeutics and fostered the design of (immuno)chemotherapy-free treatment algorithms.

## 2. *IG* Status Defines Two CLL Subsets with Different Origin and Clinical Behavior

Analysis of the tumor *IG* rearrangements revealed that CLL is divided into two major biological subsets arising at different stages of differentiation, one with unmutated *IG* (U-CLL) derived from a restricted repertoire of pre-germinal center CD5+ve B-cells and another with mutated *IG* derived from post-germinal center CD5+ve B-cells (M-CLL) [14,15,16,17,18]. The unmutated status of the expressed IG initially pointed to a naïve B-cell as the cell of origin. However, both U-CLL and M-CLL subsets are characterized by common morphological appearances and share a surface phenotype of CD5+ve activated B cells, with overexpression of the activation markers CD23, CD25, CD69, and CD71 and the underexpression of CD22, FcγRIIb (CD32b), and CD79b compared to age-matched healthy donors, and uniform expression of CD27 [19]. These characteristics are consistent with an origin of both U-CLL and M-CLL from an (auto)antigen-experienced B cell.

However, the two subsets differ in the specific expression levels of CD69, CD71, CD62L, CD40, CD39, and HLA-DR, suggesting different patterns of clinical behavior and modalities of BCR engagement. Two independent studies published back to back in 1999 revealed that U-CLL had a poorer prognosis than M-CLL [14,15]. U-CLL naturally progressed more rapidly than M-CLL, and, at the time when (immuno)chemotherapy treatment was the only approach, patients with U-CLL had shorter survival than M-CLL [14,15]. The quality of response to (immuno)chemotherapy is not dissimilar (unless TP53 defects by mutations or deletions are acquired, typically in U-CLL). However, the kinetics of progression or disease recurrence is different between the two subsets, with U-CLL recurring or progressing more rapidly than M-CLL, and this is also evident patients who have obtained undetectable levels of disease [20,21,22]. This likely reflects the distinct nature of the two subsets and a growth/proliferation signature with preferential up-regulation of *c-MYC* in the lymph node tumor cells of U-CLL rather than M-CLL [23,24].

The different origin of U-CLL and M-CLL is also reflected in their distinct DNA methylation patterns conserved at the time of transformation [25]. CpG methylation changes extensively during B cell maturation, and each stage of differentiation can be identified with a specific signature, particularly in mature B-cells [26]. Whole-genome analysis of the DNA methylome of CLL indicates that U-CLL and M-CLL maintain an epigenetic signature of which the closest normal counterparts are pre-germinal center B cells and post-germinal center memory B cells, respectively [25,27]. Although DNA methylation and gene expression appear poorly correlated, the differential methylation in the gene body has allowed the identification of a signature with functional and clinical implications for the CLL clone. The CLL with an epigenetic signature associated with U-CLL has a poor prognosis compared to those with the epigenetic signature associated with M-CLL [28].

## 3. The Clinical and Biological Significance of *IGHV3-21*-Associated Characteristics in CLL: IG Structure or Epigenetic Signature?

Although *IGHV* status is a clear indicator of prognosis, CLL using *IGHV3-21* offer an exception. Even if *IGHV3-21* is used in only ∼3% of all CLL [29], *IGHV3-21* use has been given major clinical importance due to its association with shorter overall survival (OS) independently of *IG* mutational status [30,31,32]. Around half of *IGHV3-21+ve* CLL have *IGHV(-IGHD)-IGHJ* rearrangements with unusually short *HCDR3* and non-identifiable *IGHD* sequences and are paired with *IGLV3-21* light chain [31,33]. They are enriched with mutations of the *SF3B1* gene irrespective of U-CLL and M-CLL status or CDR3 stereotypy [34] and have an epigenetic signature intermediate between U-CLL and M-CLL [25,28]. 

Initial studies aimed to answer the question of whether the specific use of *IGHV3-21* or the stereotypically short HCDR3 was linked to clinical aggressiveness, but these were in small cohorts of patients and provided conflicting results [30,31,33,35]. A subsequent analysis in a retrospective larger collection of ~430 CLL patients then suggested that the *IGHV3-21/IGLV3-21* pairing was the main determinant [36].

More recent investigations have further refined the clinical implications of *IGLV3-21* use and the associated epigenetic profile. The epigenetic signature associated with *IGV3-21* was initially described within a distinct group that had a clinical behavior “intermediate” (i-CLL) between U-CLL and M-CLL, irrespective of homology to germline *IGHV* [25,28]. There was an apparent contrast in the literature whereby *IGHV3-21* use was associated with a clinical outcome similar to U-CLL [30,31,33,35] while having an epigenetic i-CLL profile indicated an intermediate prognosis [25,28]. More recently, the use of *IGLV3-21* lambda light chain acquiring an arginine at position 110 at the link between *IGLV3-21* and *IGLC* constant region (*IGLV3-21^R110^*), or not, has been given critical importance. It now appears that the specific *IGLV3-21^R110^* usage is associated with a transcriptional profile and clinical behavior similar to U-CLL and with enrichment of *SF3B1* and *ATM* mutations. Conversely, the i-CLL lacking *IGLV3-21^R110^* rarely carry those mutations, generally express *M-IGHV*, and have a transcript profile and a clinical indolent behavior resembling M-CLL [37].

We have observed that CLL using *IGHV3-21* have high sIgM expression and signaling capacity irrespective of *IGHV* mutational status or *HCDR3* stereotypy, suggesting that *IGHV3-21* use is per se associated with sIg features of aggressive CLL (see Section 10) [38]. Our study is limited by lack of analysis of the Ig light chain and remains in a small cohort, recommending the need for further investigations. However, the sum of these studies further highlights the relevance of *IG* structure to define the biological characteristics and clinical behavior of CLL [37].

## 4. Surface IgM Dynamics Indicate Chronic Antigen Engagement in CLL

The observations that both U-CLL and M-CLL have a phenotype of activated antigen-experienced B cells and a reversible downmodulation of the sIgM support the hypothesis that CLL leukemic cells are under the chronic influence of (auto)antigen in vivo [39]. A functional description of the signaling capacity of sIgM revealed that SYK, which is recruited early following BCR engagement, was constitutively activated in both subtypes, implying (auto)antigen drive [40]. However, the most remarkable evidence of continued sIg engagement in CLL is provided by the observation that sIgM levels and responses to anti-IgM are weak and spontaneously recover following prolonged culture in an (auto)antigen free system in vitro [41]. Conceivably, temporary capping/endocytosis with anti-IgM prevents recovery, but this is reverted following anti-IgM washout.

Another feature of ongoing interaction of tumor sIg with (auto)antigen is provided by the analysis of the glycosylation of sIgM constant region heavy chain. Biosynthesis of IgM includes post-translation modifications by the addition of N-glycans to sites preserved in the heavy chain [42]. This process begins in the endoplasmic reticulum (ER), where “immature” oligomannosylated glycans are added to asparagine residues within the asparagine-x-serine/threonine acceptor sequence motifs. For the majority of normal B-cells, glycan chains are modified during transit through to the cell membrane, with further “mature” complex glycans added in the Golgi stacks at positions 171, 332, and 395 of the IgM heavy chain [43]. As a result, the main visible pattern of the sIgM of circulating resting (unstimulated) normal B-cells is one form with only mature, complex glycans. In circulating CLL cells, instead, the sIgM heavy chain exists in two forms with distinct N-glycosylation patterns. One resembles the pattern of normal circulating B cells with a mature complex type; the other is mannosylated in a manner more characteristic of immature intracellular IgM [44]. The proportion of mannosylated sIgM is variable between samples but tends to be higher in U-CLL than M-CLL. The possibility is that the biosynthetic pathway is different between the mature and immature glycoforms, but both can transduce a signal. However, the CLL pattern can be mimicked in normal B cells after ligation of sIgM. Therefore, these data provide further evidence of continued (auto)antigen engagement resulting in downmodulation of the sIgM mature form so that the mannosylated form is visible, particularly in U-CLL [44]. 

Recent studies have highlighted the possibility that CLL cells may signal through the sIg in the absence of exogenous antigenic stimulation [45]. It was proposed that the contact between the CDR3 in the heavy chain and an internal epitope of the tumor sIg would promote CLL signaling [45]. Subsequent studies also reported the presence of alternative epitopes and structural elements that can be self-recognized by the CLL BCR [46,47]. The signal emerging should turn to be homogeneous between CLL cells within individual patients and not to be affected by tissue site. However, the levels of sIgM expression and signaling capacity are variable and dynamic within the same tissue compartments of individual clones [48] and are reversible in vitro. This also happens in normal B cells following antigen engagement [41], and the critical associations of CLL outcome with the variable sIgM levels suggest that the effects of (auto)antigen engagement cannot be discounted.

## 5. *IG* Selection in CLL

Engagement of the sIg can operate either at the stage of transformation or during the maintenance and expansion of the tumor clone. The selected use of *IGHV-D-J* rearrangements, particularly prominent in U-CLL, suggests that transformation pressures operate on a restricted repertoire of normal B cells [18,49,50]. There has been a significant focus on the *HCDR3* structures to identify CLL-like sequences, and stereotypic patterns were identified [17,29]. Some of these stereotypic patterns may have a clinical impact [51], particularly those characterized by *IGLV3-21* use (see Section 4). However, the major asymmetry observed in CLL is the predominance of *IGHV1-69* (*51p1*) [52], which is used in ~30% of all U-CLL [17,29,53]. This appears tumor-related since the frequency of *IGHV1-69* in the blood of normal individuals (~1%) is very low [54]. Moreover, *IGHV1-69* rearranges with *IGHJ6* at least twice more frequently than in normal B cells [18]. The 5′ portion of *IGHJ6* extends into the *HCDR3* with a tyrosine-rich 5′ amino acid sequence that is longer and less affected by N addition/deletions than the other *IGHJs*, explaining the longer *HCDR3* and higher homology of the *HCDR3* compared to non-tumor B cells using *IGHV1-69* [18,55,56]. The same patterns can be found in a restricted repertoire of normal B cells from blood or spleen [18,57] and share several analogies with natural antibody-producing autoreactive CD27+ve B cells involved in the maintenance of immune homeostasis [39,58], by binding and clearing, e.g., apoptotic bodies, misfolded proteins, or other ubiquitous products of inflammation [59,60,61,62]. However, the preservation of a long templated unmutated *IGHV-(D-)J* rearrangement with highly asymmetric use of *IGHV1-69* and *IGHJ6* suggests that the whole variable region framework and the conserved *HCDR1-2* have an important role in both the selection and maintenance of the CLL clone (reviewed in [39]).

In M-CLL, the biased use of *IGHV-D-J* rearrangements is less apparent, and *IGHV4-34*, which is the most common in CLL, is used as frequently as in the normal B-cell repertoire. This may reflect a different modality of BCR engagement (reviewed in [39]), ultimately affecting tumor behavior.

## 6. Surface Ig Engagement Occurs at Tissue Sites in CLL

The role of the microenvironment in CLL has long been sought by investigations in vitro [63,64,65], mainly due to difficulties in accessing material from tissues other than the peripheral blood. Gene expression profiling of matched blood, bone marrow, and lymph node CLL cells has identified the lymph node as a key site where the BCR is activated [23]. The activation signature is associated with increased SYK phosphorylation and higher proliferation [23]. 

The existence of sIg engagement at tissue sites has also been implied by the observation of the changes of expression levels and glycosylation patterns of the sIgM on the circulating CLL cells of patients during therapy with ibrutinib [66]. Daily ibrutinib therapy inhibits Bruton’s tyrosine kinase (BTK)-dependent signaling and redistributes leukemic cells from tissue to blood. This effect of ibrutinib therapy is caused by the inhibition of chemokine receptors such as CXCR4 and adhesion molecules, which also utilize BTK for signaling [67,68]. Consequently, when patients with CLL receive long-term therapy with daily ibrutinib doses, tissue-homing functions are inhibited, and CLL cells are “drifted away” into the blood and cannot re-enter tissue [69,70]. During this time of continued ibrutinib therapy, CLL cells are deprived of tissue-based microenvironmental stimuli, and proapoptotic and autophagocytic mechanisms kick on: CLL cells increase expression of proapoptotic Bim_EL,_ and LC3B-II promotes autophagocytosis while shrinking in size and downmodulating expression of the majority of surface receptors, including sIgD [66]. Despite these events, the same cells have a significant increase in sIgM expression. The specific increase in sIgM on the CLL cells of the peripheral blood during ibrutinib is strongly indicative of recovery from antigen-mediated downmodulation. Disengagement from antigen is also documented by the conversion of the sIgM glycosylation pattern from immature to mature and lack of further increase in sIgM expression during subsequent culture in vitro [44,66]. The specific increase and conversion from immature to mature conformation of sIgM in the circulating CLL cells under ibrutinib underpin the key role of engagement of CLL cells with antigen located in tissues, likely the lymph node.

## 7. CLL at the Edge between Anergy and Survival

The substantial reduction in sIgM expression and signaling capacity of the circulating leukemic cells is a remarkable peculiarity of CLL [1,71]. This feature distinguishes CLL from the normal B cells and other mature B-cell tumors that typically express high levels of sIg and mediate strong intracellular signals following anti-Ig stimulation in vitro. The reduction in sIgM expression signaling capacity is a functional state that resembles the anergic anti-HEL (Hen Egg Lysozyme) B cells of mouse models producing HEL [72]. In these mouse models, anti-HEL B-cells are in a state of sIgM, but not sIgD, non-responsiveness, and inability to induce proliferation signals. When anti-HEL B cells are transferred into a non-HEL mouse, they specifically regain sIgM levels and function. This reversible state of “anergy”, which appears designed to maintain tolerance to self and prevent autoreactive B cells from mounting an autoimmune response while remaining responsive to foreign antigens [71,72,73,74], is imitated by CLL for many characteristics. Like anergic B cells, CLL cells have raised basal intracellular Calcium (iCa^2+^) [71], reduced differentiation capacity [48,75], increased NFAT expression and ERK1/2 phosphorylation [76,77], and increased expression of the proapoptotic Bim isoforms, Bim_EL_ and Bim_L_ [78]. Another link with anergy is the ability of CLL cells to produce IL10. We observed that the production of IL10 is variable in CLL and that variability is closely associated with the grade of anergy and with differential *IL10* gene methylation [79].

However, anergic B cells are generally short-lived to avoid undesirable autoimmune reactions [73]. In contrast, CLL cells are notoriously long-lived. The main explanation for this is the overexpression of Bcl-2 in CLL [80,81,82]. Bcl-2 plays a fundamental antiapoptotic role in any B cell [83]. The large majority of CLL cells have a deletion of the *13q14* locus, which involves at least the *miR-15a/16-1* locus [81]. These microRNAs function by repressing Bcl-2 expression, and their deletion leads to very high Bcl-2 protein levels [80]. In this context, anergy, which should favor apoptosis, is counterbalanced by the constitutive overexpression of Bcl-2. This appears clinically important and might explain why CLL, and not other B-cell tumors that do not have anergy, is highly sensitive to BH3-mimetics, such as venetoclax [84]. Sequestration of Bcl-2 by venetoclax sets CLL cells free from antiapoptotic constraints allowing proapoptotic molecules (such as Bim) to tilt the anergic CLL cells towards death [78].

## 8. Microenvironmental Influences on sIgM Expression and Function

**Figure 1:**
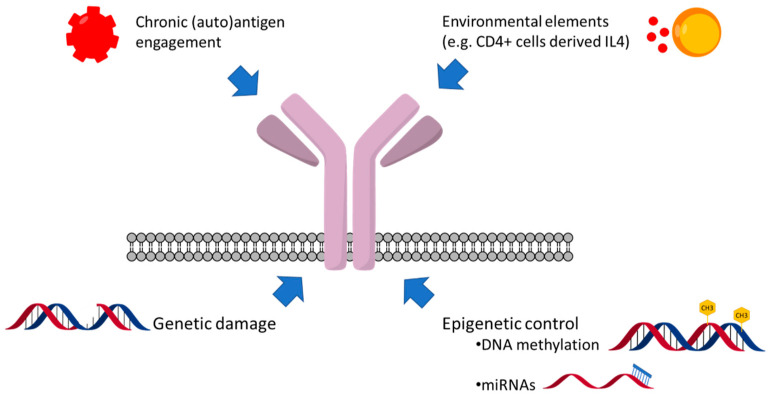
Microenvironmental and (epi)genetic factors influencing surface IgM levels and signaling capacity in CLL.

While (auto)antigenic drive operates at tissue sites, other factors appear as important potential determinants of sIg responses (Figure 1). Several environmental, cellular, and soluble elements affect CLL behavior [39], and, unlike what is typically seen in the peripheral blood, a small fraction of CLL cells overcomes anergy and proliferates in the lymph node. Although T-cell defects are apparent in CLL and there is no clear evidence of cognate T-cell help [85,86,87], this would allow escape from anergy [88]. CD4+ve T cells, which are one of the main sources of IL4, are present near survivin+ve, Bcl-2+ve leukemic B cells in the proliferating centers of CLL [89,90]. A comparison of lymph nodes with peripheral blood cells showed that the mean sIgM levels in U-CLL cells of the lymph node are higher than in the peripheral blood [91]. This may suggest that the balance between antigen-mediated endocytosis and cytokine-induced sIgM upregulation is skewed towards the latter to allow the proliferation of the fraction of cells that have overcome the anergy threshold. Of all cytokines tested in vitro, IL4 and, in part, autocrine IL6 upregulate sIgM expression and function in CLL [92,93,94]. This may be clinically important since IL4-induced sIgM upregulation also appears to confer partial resistance to ibrutinib in vitro and offer the rationale for therapies with dual JAK/SYK inhibitors [92,95].

## 9. Genetic and Epigenetic Factors Affecting sIgM Expression and Function

Intrinsic factors contribute further to the heterogeneity of U-CLL and M-CLL. Our data indicate an association of genetic lesions predicting high-risk disease, or DNA methylation status, with sIgM levels/signaling predominantly in U-CLL, and M-CLL, respectively [38]. 

In U-CLL, we found that trisomy *12* and genetic lesions at *17p/TP53* locus, but not isolated deletion of *13q* or deletion of *11q* locus, are associated with increased sIgM expression and signal capacity [38]. However, trisomy *12* is also enriched with CD49d expression and up-regulation of integrin signaling, *NOTCH1* mutation, or *IGHV4-39* use. These features are individually claimed as independent factors of aggressive disease [96,97,98,99], and it is difficult to understand if these lesions are simple associates or also explain the variations in sIgM. However, in U-CLL that do not carry trisomy *12*, sIgM expression and signaling capacity are higher when NOTCH1 activity is stabilized by mutations in the PEST domain [38]. In these U-CLL, pre-treatment of tumor cells with γ-secretase inhibitor (DAPT) decreases sIgM levels and signaling capacity, suggesting that the increased BCR signaling capacity is also supported by NOTCH1 stabilization [100]. Potent sIgM signaling also induces NOTCH1 protein levels via direct MYC-dependent regulation of the global translation machinery [101,102], which per se can be a therapeutic target [103,104]. Where further potentiated by NOTCH1 activation, the sIgM mediated protein translation is also suppressed by DAPT [100]. These data suggest that a specific genetic lesion can directly affect sIgM expression and function, whereby specific stabilizing *NOTCH1* mutations contribute to increasing the sIgM expression and function in the CLL cell.

In M-CLL, where there are fewer high-risk genetic lesions, analysis of the epigenetic signature revealed a strikingly high correlation between the maturation of DNA methylation and the reduction in sIgM levels or signaling capacity [27,38]. It appears that the CLL cells derived from more mature B cells within the spectrum of M-CLL may be more susceptible to induction of anergy. Whilst it is not known if DNA-methylation directly influences sIgM per se, these results suggest that variable sIgM responsiveness may in part be also an intrinsic feature, dependent on the cell of origin and potentially influenced by DNA-methylation.

*Micro-RNAs (miRs)* can also negatively or positively regulate antigen-dependent and antigen-independent function of the tumor sIg [105]. Their expression profile is different in U-CLL compared to M-CLL [106], possibly resulting from both intrinsic characteristics and microenvironmental influences of different types in the two subsets, and is associated with diverse prognoses [82]. *MiR-155*, which can be induced by CD40L or BAFF, is associated with more aggressive disease, particularly in patients with M-CLL, and enhances BCR signaling following anti-Ig ligation, whereby miR-155 inhibitors block this effect [107]. Conversely, *miR-150*, which is less expressed in U-CLL than in M-CLL and inversely correlates with disease progression and overall survival, appears instead to regulate BCR signaling negatively. High-level expression of *miR-150* can repress forkhead box P1 (FOXP1) and GRB2-associated binding protein 1 (GAB1), which encode proteins that enhance anti-IgM-mediated and constitutive BCR signaling [108,109]. Clearly, other miRs, including *miR-17-92, miR-181, miR-29, or miR-34,* can regulate the activated phenotype of CLL cells or function of the sIg [105,110], further explaining disease heterogeneity.

## 10. The Consequences of the Variable sIgM Expression Levels and Function on CLL Progression

Assignment of CLL to the U-CLL or M-CLL subset is now becoming a necessity for risk stratification algorithms. International multicenter meta-analyses identified the *U-IGHV* status as one of the major independent factors conferring a higher risk of progression of either asymptomatic early-stage CLL or any other CLL at diagnosis or following (immuno)chemotherapy [20,111,112].

However, the simple measurement of sIgM levels by phenotypic analysis and/or function by iCa^2+^ mobilization assay in vitro can reveal the clinically relevant heterogeneity of CLL by conveying microenvironmental, epigenetic, and genetic influences on CLL into one parameter [38]. These factors are distributed differently between U-CLL and M-CLL [38]. 

The sIgM (but not sIgD) expression and competence to respond to anti-IgM are generally higher in U-CLL than in M-CLL [40] and are independent parameters predicting progression to first treatment [38]. In a multivariate analysis, we found that the role of sIgM levels is independent of known phenotypic, genetic, or methylation prognostic markers of progression. An independent study has confirmed this observation and explained the most aggressive outcome of CLL with high sIgM compared to low sIgM by documenting that sIgM, but not sIgD, levels are associated with CLL cell birth rates, suggesting the higher sIgM levels (but not sIgD) associate with cell growth and metabolic activity [113]. However, the variability of sIgM levels and signaling capacity are evident even within each of the two U- and M-CLL subsets. Their measurement allows the subdivision in additional sub-categories of U-CLL with high or low sIgM, or M-CLL with high or low sIgM, where U-CLL with high sIgM has the worse prognosis, and the M-CLL with low sIgM has the best [38]. 

Although the measurement of sIgM levels requires standardization of protocols and validation in independent cohorts [114], the published data claim the potential clinical utility of sIgM to identify those CLL with more aggressive behavior.

## 11. Surface IgM Levels and Function May Identify Responses to BCR Inhibitors in Patients with CLL

**Figure 2:**
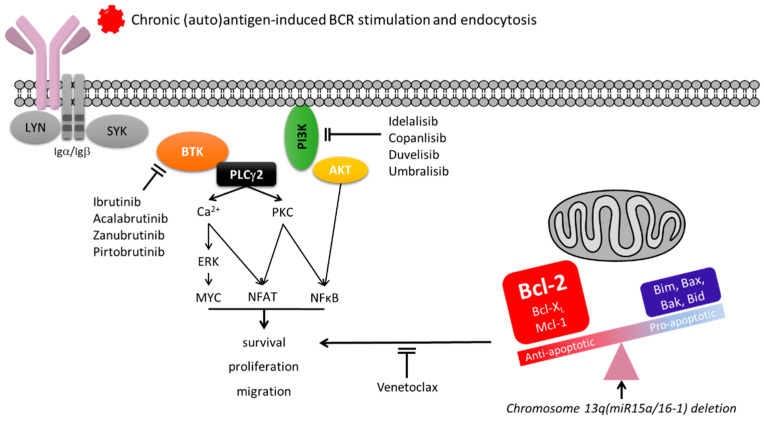
Simplified diagram of BCR signaling and therapeutic targeting in CLL. BCR engagement by (auto)antigen leads to the proximal activation of a complex of kinases and scaffold proteins, initiating with the phosphorylation of the immunoreceptor tyrosine-based activation motifs (ITAMs) in the C-terminal tail of BCR-associated Igα/CD79a and Igβ/CD79b by LYN. Phosphorylated ITAMs leads to SYK recruitment and propagation of signal to Bruton tyrosine kinase (BTK) and phospholipase Cγ2 (PLCγ2). LYN-dependent phosphorylation of the cytoplasmic domain of CD19 also recruits phosphoinositide 3-kinase (PI3K). Activation of a network of distal signaling molecules follows. Activation of PLCγ2 leads to the release of intracellular Ca2+ and activates protein kinase C (PKC). PKC subsequently induces the activation of transcription factors, including NF-κB and nuclear factor of activated T cells (NFAT). Recruitment of PI3K to the plasma membrane leads to optimal activation of BTK and AKT. PLCγ2 is also involved in the activation of mitogen-activated protein kinase (MAPK) pathways, including the extracellular signal-regulated kinase 1/2 (ERK). The third phase of events involves modulation of multiple downstream regulators, which ultimately mediate changes in cell proliferation, survival, and migration, via both phosphorylation and transcriptional modulation of key regulators of cell survival (e.g., Mcl-1, Bim). In CLL, strength of BCR signal, which is controlled by surface IgM levels, will determine cell fate with a balance towards anergy particularly when sIgM levels are low. MiR15a/16-1 deletion at chromosome 13, allowing overexpression of antiapoptotic Bcl-2 protein favoring survival, counter the proapoptotic mechanisms associated with anergy. Therapeutical inhibition of BTK variably blocks BCR signaling in CLL depending, amongst other factors, on sIgM levels and signal strength. Addition of PI3K inhibitors may fully suppress the residual signaling activity. BH3 mimetics (venetoclax) block the antiapoptotic mechanisms resulting from Bcl-2 overexpression found in CLL.

BCR-associated kinase inhibitors are very effective in CLL. Clinical use is skewed towards BTK pathway inhibitors (BTKi) (Figure 2), amongst which ibrutinib was the first in kind to rapidly shift medical algorithms away from chemotherapy [115,116,117]. Following their introduction in the clinical practice, the status of the tumor *IGHV* has become a determinant for the best treatment choice. Clinical trials comparing the efficacy of immunochemotherapy versus BTKi ibrutinib or acalabrutinib have revealed that, while the duration of response is generally not dissimilar in M-CLL, there is a remarkable benefit on progression-free survival in patients receiving BTKi compared to immunochemotherapy in U-CLL [118,119].

However, CLL can develop resistance to BTKi. The occurrence of genetic lesions, including mutations of the *BTK* and *PLCG2*, have been frequently documented in the CLL cells of these patients [120,121]. These mutations are subclonal [121,122,123,124,125,126,127,128,129] and appear insufficient to keep the signaling pathway active unless there is also sIgM engagement [120], suggesting that this is also necessary for evasion from ibrutinib. Indeed, the CLL cells that redistribute and survive in the peripheral blood during continued therapy with ibrutinib selectively recover and maintain functionally competent IgM on the cell surface [66]. The signaling potentials of those cells appear specifically dependent on the sIgM levels [66]. During this time in the circulation, the CLL cells with high sIgM appear to develop adaptation mechanisms for survival, including an increase of constitutive AKT phosphorylation [130,131], which may result from the induction of the FoxO1-GAB1 axis [108]. High surface IgM may instead provide a proliferation advantage on those residual “dangerous” cells equipped to reach tissue sites [48]. We found that high sIgM levels of expression and signaling capacity correlate with a shorter duration of response to ibrutinib and a faster disease progression. Despite the ability to fully occupy and inhibit BTK, CLL cells appeared not to completely inhibit sIgM-mediated iCa^2+^ and ERK phosphorylation downstream to BTK if the sIgM expression and signaling capacity are strong. These results confirm that sIgM signaling is dependent on sIgM levels and can circumvent BTK blockade when sIgM levels are high [131].

An approach currently used in clinical trials to increase efficacy and reduce the risk of resistance is the combination of a BTKi and a BH3 mimetic. This combination using ibrutinib and venetoclax is revealing a dramatic efficacy in patients with either treatment-naïve or relapsed/refractory CLL [132,133]. However, while ibrutinib will synergize with the BH3 mimetic venetoclax, there is always a risk that antiapoptotic molecules, including Mcl-1, which is regulated via a canonical AKT-dependent pathway [134], may not be completely suppressed. The sIgM levels may indirectly inform the degree of AKT activation and, while clinical trial data are maturing with longer follow-ups, it may be possible that different combinations including PI3K/AKT inhibitors may need renewed consideration in CLL with high sIgM.

## 12. Conclusions

**Figure 3:**
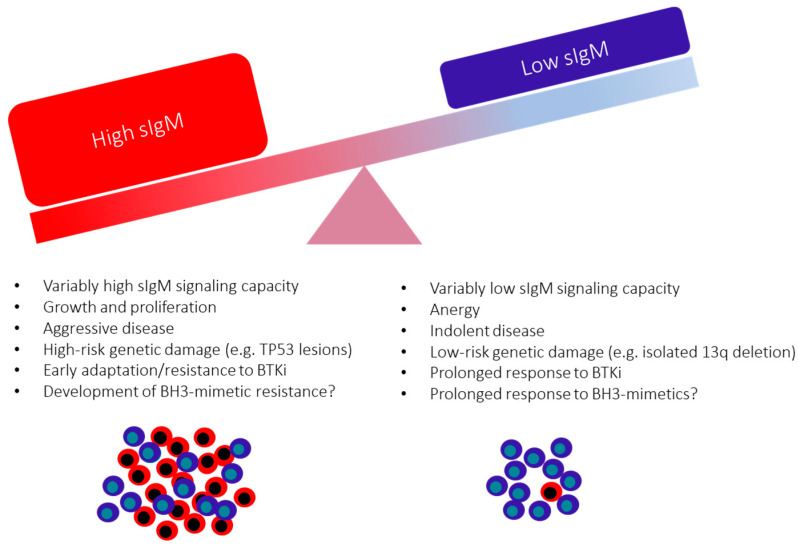
The variable consequences of surface IgM levels on CLL behavior and clinical outcome.

Investigations of the sIg structure and function have continued to provide fundamental insight into CLL biology and refinement of therapy. U-CLL and M-CLL are characterized by a variable degree of anergy, defined by low sIgM levels and reduced signaling capacity consequent to chronic engagement by cross-reacting (auto)antigens [73]. Although microenvironmental and genetic features also influence this, the low sIgM expression and function are, in part, reversible in vitro and during circulation in blood [41,48,66]. The consequences of the variably low sIgM levels and signaling are variable growth, proliferation, and survival of individual tumor cells [102,135] sustained by constitutive and IgM-induced antiapoptotic mechanisms (Figure 3) [71,136,137]. These can now be co-targeted by BTKi and BH3- mimetics [132,133], and several phase 3 clinical trials have been activated to investigate the efficacy and toxicity of this combination (Table 1). Particular attention should be given to those CLL cells where the increased sIgM signaling may favor BTK blockade by-pass and eventually lead to therapy resistance of the less anergic CLL cells [131]. The prognostic and therapeutic value of sIg analysis indicates the importance of continuing to analyze sIg levels and structure to refine the understanding of the origin, maintenance, progression, therapy, and prognosis of U-CLL and M-CLL.

## Figures and Tables

**Table 1:** Phase 3 clinical trials currently registered at Clinicaltrials.gov using a BTKi with a BH3-mimetic in CLL (January 2022).

NCT Number	Investigation	Comparator(s)	Setting	Status	Main Outcome Measures	Enrollment	Sponsor	Start	Completion
NCT05057494	Acalabrutinib + Venetoclax	Venetoclax + Obinutuzumab	Untreated	Not yet recruiting	PFS; PB and BM uMRD; OS; EFS; ORR; CR rate; QLQ; AE	750	Industry	Mar-22	Sep-28
NCT04965493	Pirtobrutinib + Venetoclax + Rituximab	Venetoclax + Rituximab	relapsed/refractory	Recruiting	PFS; OS; TTNT; EFS; ORR	600	Industry	Sep-21	Oct-25
NCT04608318	Ibrutinib + Venetoclax	Venetoclax + Obinutuzumab or Ibrutinib	Untreated	Recruiting	PFS; PB and BM uMRD; ORR; CR; AE	897	Academic	Mar-21	Mar-27
NCT03836261	Acalabrutinib + Venetoclax ± Obinutuzumab	FCR or BR	Untreated	Recruiting	PFS	780	Industry	Feb-19	Jan-27
NCT03737981	Ibrutinib + Venetoclax + Obinutuzumab	Ibrutinib + Obinutuzumab	untreated, elderly	Recruiting	PFS; BM MRD; CR rate; OS; AE	454	Academic	Jan-19	Jun-27

PFS: progression-free survival; PB: peripheral blood; BM: bone marrow; uMRD: undetectable minimal residual disease; OS: overall survival; EFS: event-free survival; ORR: Overall Response Rate; CR: complete response; QLQ: quality of life questionnaire; AE: adverse events; TTNT: Time to next treatment; FCR: fludarabine, cyclophosphamide, rituximab; BR: bendamustine, rituximab.
